# Clinical characteristics of pneumothorax and pneumomediastinum in mechanical ventilated patients with coronavirus disease 2019: a case series

**DOI:** 10.1186/s13256-023-04281-6

**Published:** 2024-01-02

**Authors:** Yohei Ide, Nao Urushibata, Wataru Takayama, Kenichi Hondo, Junichi Aiboshi, Yasuhiro Otomo

**Affiliations:** Trauma and Acute Critical Center, Tokyo Medical and Dental University Hospital of Medicine, 1-5-45, Yushima, Bunkyo-ku, Tokyo, 113-0034 Japan

**Keywords:** COVID-19, Intensive care units, Intubation, Pneumothorax, Case series

## Abstract

**Background:**

Pneumothorax (PTX) and pneumomediastinum (PM) have been reported as potential complications in patients with coronavirus disease 2019 (COVID-19); however, their risk factors and etiology remain unknown. Herein, we investigated the clinical characteristics of mechanically ventilated patients with COVID-19 with PTX or PM.

**Methods:**

We examined patients with severe COVID-19 requiring mechanical ventilation who were admitted to the intensive care unit of a tertiary-level emergency medical center in Tokyo, Japan between April 1, 2020. and October 31, 2021. We collected and analyzed the clinical characteristics of the patients who presented with either PTX or PM during mechanical ventilation.

**Results:**

During the study period, a total of 165 patients required mechanical ventilation, and 15 patients with PTX/PM during mechanical ventilation were selected. Three patients with obvious causes were excluded, and the remaining 12 patients were analyzed (7.3%). The mortality rate in these patients was as high as 50%, demonstrating the difficulty of treatment in the presence of PTX/PM. PTX/PM occurred 14.5 days after intubation. A peak pressure of > 30 cmH_2_O was only apparent in one patient, suggesting that high positive pressure ventilation may be less involved than mentioned in the literature. In addition, the inspiratory effort was not strong in our group of patients. (P0.1 was 2.1 cm H2O [1.0–3.8]).

**Conclusion:**

Various factors are associated with the development of PTX/PM in patients on mechanical ventilation for COVID-19. We did not find a strong correlation between PTM/PM and barotrauma or strong inspiratory efforts, which have been identified as potential causes in previous studies.

## Background

Coronavirus disease 2019 (COVID-19) is a global pandemic since first identified in China in December, 2019 [1]. Pneumothorax (PTX) and pneumomediastinum (PM) are possible complications during the course of COVID-19; retrospective studies of patients with COVID-19 have suggested that PTX may occur in 1% of those requiring hospital admission and in 2% of patients requiring intensive care unit (ICU) admission [2]. Although the underlying pathological mechanism is not fully understood, PTX/PM may be caused by barotrauma due to high positive pressure ventilation during mechanical ventilation and loss of lung compliance due to the significant alveolar damage [3]. However, barotrauma alone is unable to explain the development of PTX/PM completely [4]. Previous studies suggest that multiple factors such as the presence of lung diseases, smoking history, steroid use, and cytokine storm of severe COVID-19 are involved [5–7]. However, risk factors and etiology regarding the formation of PTX/PM remain unclear. PTX was associated with a significant increase in patient mortality (66% vs. 46%) [8].

Therefore, we investigated the clinical characteristics of PTX/PM, focusing on patients with severe COVID-19 requiring mechanical ventilation who were admitted to the ICU.

## Methods

### Patient population

This case series was conducted in the ICU of a tertiary-level emergency medical center in Tokyo, Japan. Patients with COVID-19, who were admitted to the ICU of the Tokyo Medical and Dental University Hospital (Tokyo, Japan) between April 1, 2020 and October 31, 2021 and required mechanical ventilation, were reviewed in this study. A diagnosis of COVID-19 was determined for all patients based on findings of the nasopharyngeal swab test for severe acute respiratory syndrome coronavirus 2 (SARS-CoV-2) by using real-time reverse transcriptase polymerase chain reaction. We included all patients with COVID-19, who required mechanical ventilation and developed PTX/PM during ICU treatment. PTX/PM was diagnosed by chest radiograph or computed tomography (CT). We excluded patients with obvious causes of PTX/PM, such as after the insertion of a jugular venous catheter. This case series was approved by the Institutional Review Board of our institution (M2022-001).

### Patient management

Patients with COVID-19 received mechanical ventilation if oxygenation with conventional oxygen therapy could not be maintained. The patients were considered for prone position therapy (PPT) if they could not maintain a PaO_2_/FIO_2_ (P/F) ratio of 200 during mechanical ventilation. If patients were unable to maintain a P/F ratio of 100–150 after PPT, venovenous extracorporeal membrane oxygenation (VV-ECMO) was considered. However, there were no definite criteria, and the indication was discussed and decided upon in a multi-professional conference, considering factors such as age, lung condition, duration of treatment, underlying disease, and general condition. We defined acute respiratory distress syndrome (ARDS) according to the Berlin definition [9].

The standard treatment for COVID-19 at our hospital includes the administration of 6.6 mg dexamethasone for 7–10 days and remdesivir for 10 days. In addition, in cases where respiratory failure progresses rapidly, pulse methylprednisolone therapy (1000 mg methylprednisolone for 3 consecutive days) may be selected at the discretion of the attending physician.

### Data collection

The following data were collected retrospectively from medical records:

age; sex; body mass index (BMI); comorbidities; smoking history; chest radiograph and CT; treatment for COVID-19; the administration of VV-ECMO and PPT: complications; management for PTX/PM; status on hospital discharge (i.e., dead or alive); and the length of ICU stay. We also collected mechanical ventilation parameters (e.g., positive end-expiratory pressure (PEEP), plateau pressure, peak pressure, and inspiratory effort). Inspiratory effort was measured using airway occlusion pressure (P0.1), defined as the negative pressure measured at the airway opening 100 ms after the initiation of inspiratory effort, performed against a closed respiratory circuit [10].

## Results

**Fig. 1:**
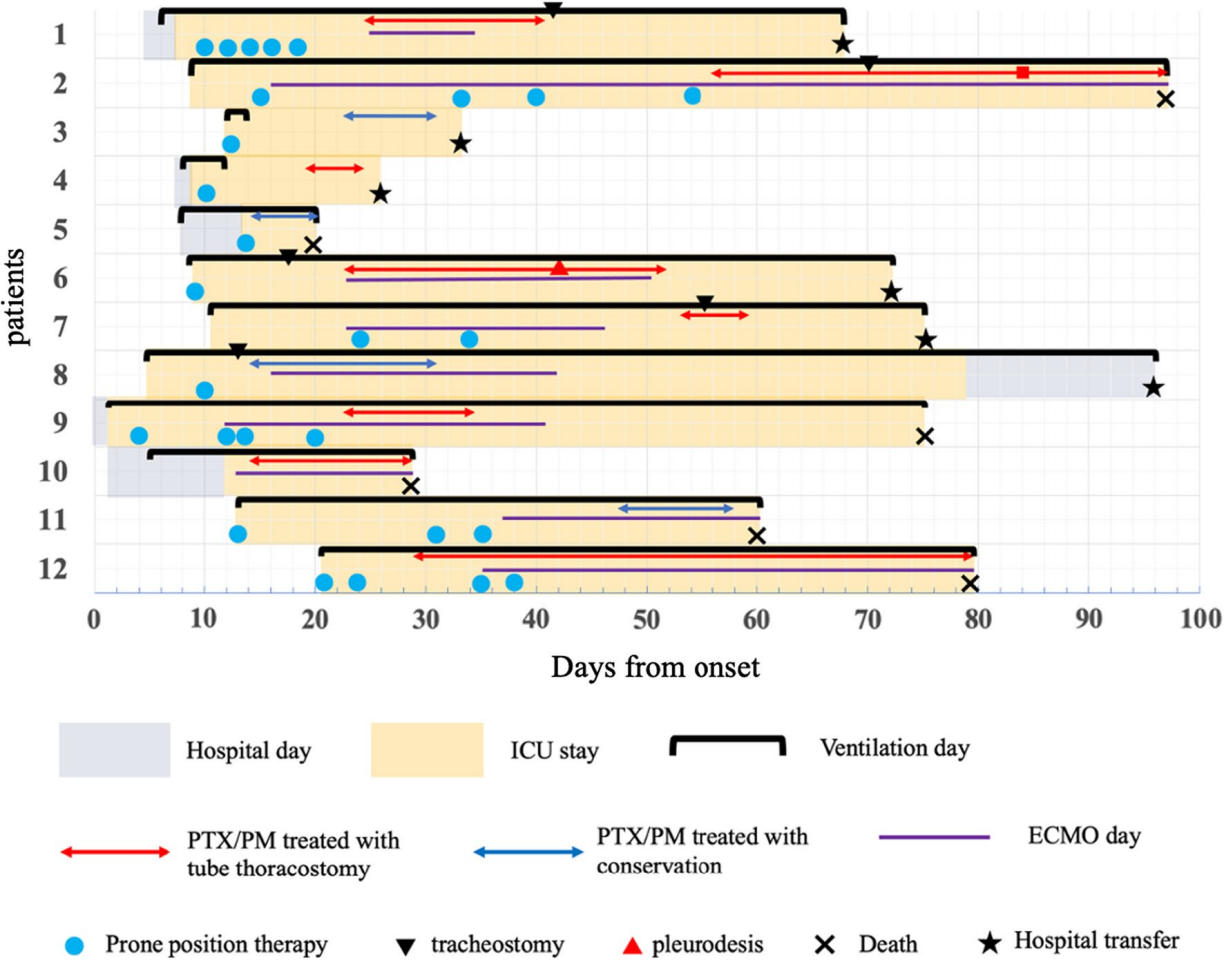
Patients’ course of events from admission to discharge. We analyzed 12 patients with COVID-19, who required mechanical ventilation and developed PTX/PM during ICU treatment. *PTX* pneumothorax, *PM* pneumomediastinum, *ICU* intensive care unit, *COVID-19* coronavirus disease 2019

**Fig. 2:**
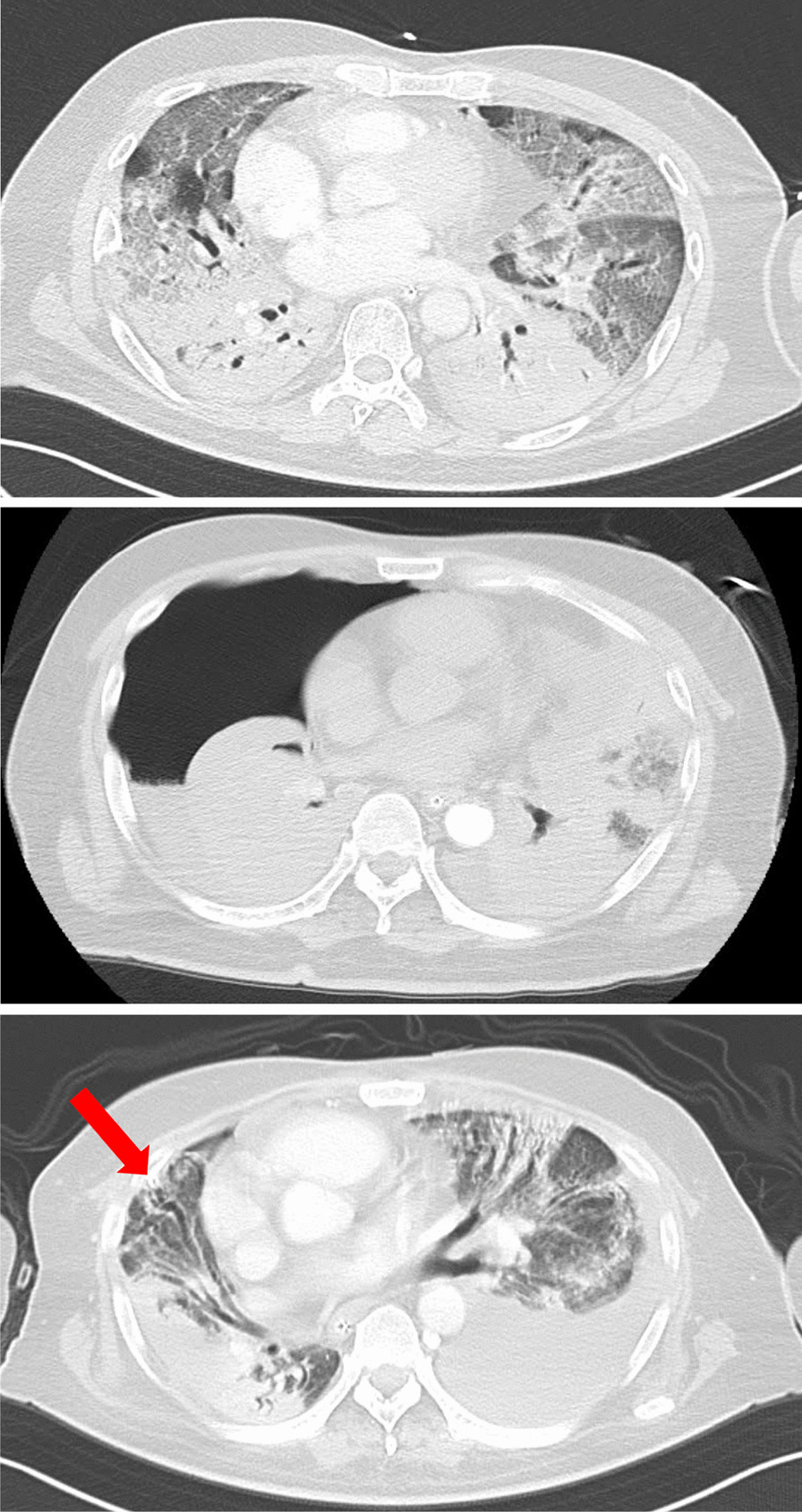
Imaging course of one patient demonstrating PTX without PM, caused by COVID-19. Imaging course at the time of admission, at the onset of PTX, and after improvement. The patient showed only right PTX and was treated with tube thoracotomy (arrow). *PTX* pneumothorax, *PM* pneumomediastinum, *COVID-19* coronavirus disease 2019

In total, 667 patients diagnosed with COVID-19 were admitted to our hospital during this period, of whom 165 were intubated. Fifteen patients developed PTX/PM, and three were excluded based on the exclusion criteria. The remaining 12 patients were included in the final analysis. Patients’ course of events is shown in Fig. 1. The detailed characteristics of the population are presented in Table 1 and imaging course of one patient at the time of admission, at the onset of PTX, and after improvement are shown in Fig. 2. Among these patients, three (25%) presented with both PTX/PM, seven (58%) with PTX alone, and two (17%) with PM alone. The overall incidence of PTX/PM in mechanically ventilated patients was 7.3%. PTX/PM occurred 23 days (17–38.5) after the onset of COVID-19 and 14.5 days (10–28.5) after intubation. All patients with PTX/PM at our hospital were male and were unvaccinated. Regarding underlying diseases, hypertension was the most common comorbidity with seven (58%) patients, followed by diabetes mellitus (25%) and chronic kidney disease (17%). One patient had a past history of asthma, but the remaining 11 patients (92%) had no history of any pulmonary disease. One patient showed emphysema on chest CT at admission, whereas the remaining 11 patients showed no such findings at admission. However, during the course of ICU treatment, three patients developed a new bulla, which was detected on chest CT before the occurrence of PTX/PM. In many cases, the first symptoms when they were diagnosed with COVID-19, were fever (92%) and dyspnea (83%).

**Table 1:** Comparison of medical characteristics of patients

Patient	Age	Sex	BMI	PTX	PM	From onset to occurrence PTX/PM, in days	From mechanical ventilation to occurrence PTX/PM, in days	The first symptom				Smoking history	Respiratory comorbidities	Other comorbidities				CT features on admission
								Fever	Dyspnea	Diarrhea	Dyspnea			HT	DM	CKD	Malignancy	
1	57	M	24.1	+	−	25	19	+	+	−	−	−	Asthma	+	−	−	−	None
2	66	M	24.3	+	−	56	47	+	+	+	−	+	None	+	−	−	−	None
3	76	M	28.7	+	+	22	10	+	+	−	+	−	None	−	−	−	+	None
4	56	M	27.2	+	−	19	10	+	+	−	−	+	None	+	+	−	−	None
5	94	M	19.9	−	+	15	7	+	+	−	−	−	None	+	-	+	−	None
6	74	M	26.6	+	−	23	14	+	+	−	−	−	None	+	−	−	−	Emphysema
7	54	M	34.4	+	−	53	35	+	+	−	+	+	None	+	−	+	−	None
8	70	M	22.3	+	+	14	10	+	-	−	−	−	None	−	−	−	−	None
9	66	M	26.2	+	−	23	22	-	+	−	−	−	None	−	−	−	−	None
10	62	M	28.4	+	−	12	7	+	+	−	−	−	None	+	+	−	−	None
11	54	M	25.1	−	+	48	35	+	-	−	−	+	None	−	+	−	−	None
12	58	M	26.7	+	+	29	15	+	+	−	−	−	None	−	−	−	−	None
Median	64		26.4			23[17–38.5]	14.5[10–28.5]											
[IQR]	[56.5–72]		[24.2–27.8]															

In this study, three patients (25%) presented with both PTX/PM, seven patients (58%) with PTX alone, and two patients (17%) with PM alone. All patients were male. Only one patient had respiratory comorbidities, and only one patient showed emphysema on chest CT at admission

Continuous variables are expressed as medians (IQR)

*BMI* body mass index, *PTX* pneumothorax, *PM* pneumomediastinum, *CT* computed tomography, *IQR* interquartile range, *HT* hypertension, *DM* diabetes mellitus, *CKD* chronic kidney disease

The treatment options for COVID-19 are presented in Table 2. Steroids were administered to 11 patients (91.7%). Six patients presented with PTX/PM after completion of the 10 days dexamethasone 6.6 mg treatment. Four patients were administered pulsed methylprednisolone. The treatment variation was partly due to treatment uncertainty during the early stages of the pandemic. Forty intubated patients underwent steroid pulse therapy at our hospital, and PTX occurred in four patients (10%).

**Table 2:** Treatment, complications, and outcome of COVID-19, and management of PTX/PM

	Treatment						Complications		Outcome	
	Dexamethasone 6.6 mg × 7–10 days	Methylprednisolone 1000 mg	ECMO day from PTX/PM	PPT	No of PPT before PTX/PM	Onset day after last PPT	ARDS	Management of PTX/PM	Death	ICU length of stay, in days
1	−	−	0	+	5	6	+	Tube thoracotomy	−	60
2	−	+	50	+	4	1	+	Tube thoracotomy + bronchial occlusion	+	88
3	+	−	−	+	1	8	−	Conservative	−	21
4	−	+	−	+	1	7	+	Tube thoracotomy	−	17
5	+	−	−	+	1	0	−	Conservative	+	6
6	+	−	I0	+	1	2	−	Tube thoracotomy + pleurodesis	−	63
7	+	−	29	+	2	17	+	Tube thoracotomy	−	64
8	+	+	-2	+	1	2	+	Conservative	−	75
9	+	−	11	+	4	1	+	Tube thoracotomy	+	40
10	+	−	-1	−	-	-	+	Tube thoracotomy	+	17
11	+	−	11	+	3	12	+	Conservative	+	47
12	+	+	-6	+	4	3	+	Tube thoracotomy	+	59
Median (IQR)					2 (1–4)	3 (1–8)				53 (19–63.5)

Steroids were administered to 11 (91.7%) patients to treat COVID-19. Two patients had PM only and were treated conservatively. Of the 10 patients with PTX, eight were treated with tube thoracostomy, and two were managed conservatively. The mortality rate of patients with PTX or PM was 50%

Continuous variables are expressed as medians (IQR)

*COVID-19* coronavirus disease 2019, *ARDS* acute respiratory distress syndrome, *ECMO* extracorporeal membrane oxygenation, *PTX* pneumothorax, *PM* pneumomediastinum, *PPT* prone position therapy, *ICU* intensive care unit, *IQR* interquartile range

The mechanical ventilation settings before the occurrence of PTX/PM are shown in Table 3. Three cases occurred during VV-ECMO, although none were in lung rest settings and were managed under the same lung-protective settings recommended for ARDS (tidal volume set to 6–8 mL/kg to the predicted body weight). Regarding ventilator settings, PEEP was set at 11 cmH2O (10–12), plateau pressure was 15 cmH2O (12.5–17.5), and peak pressure was 24.5 cmH2O (20.5–26.5). None of these pressures were very high. Respiratory effort was never strong; recorded P0.1 was 2.1 cmH2O (1.0–3.8).

**Table 3:** Mechanical ventilator settings before occurrence of PTX/PM

	Mode	PEEP set, cmH2O	Plateau pressure, cmH2O	Peak pressure, cmH2O	P0.1 cmH2O ^†^	PaO2/FIO2	PaCO2	Crs
1	A/C	10	21	21	ー	189	55.7	38.9
2	A/C on ECMO	15	27	27	0.5	273	34.3	15
3	CPAP + PS	10	15	15	8	138	35.1	104
4	CPAP + PS	8	14	14	1.7	207	53.9	71.2
5	A/C	10	29	29	ー	290	47.1	26.8
6	CPAP + PS	10	20	20	1	168	36.8	60.4
7	CPAP + PS	8	26	26	0.9	285	45.3	37.6
8	CPAP + PS	12	23	23	2.6	119	37	62.8
9	A/C on ECMO	12	32	32	3.8	249	46.3	50
10	A/C	12	22	22	2.1	91	44.1	55.7
11	A/C on ECMO	12	26	26	7.3	144	48.5	22
12	A/C	12	26	26	2	184	48.8	38.9
		11(10–12)	15 (12.5–17.5)	24.5 (20.5–26.5)	2.05 (1.0–3.8)	185.5 (140.9–260.8)	45.8 (36.9–48.7)	44.5 (32.2–61.6)

Three cases occurred during VV-ECMO, although they were managed under the same lung-protective settings, not lung rest settings, as recommended for ARDS. Peak pressure was 24.5 cmH2O (20.5–26.5). A high peak inspiratory pressure of 40–50 cmH2O was not detected. Respiratory effort was never strong; recorded P0.1 was 2.1 cm H2O (1.0–3.8)

Continuous variables are expressed as medians (interquartile range)

*A/C* assist/control, *VV-ECMO* venovenous extracorporeal membrane oxygenation, *CPAP* continuous positive airway pressure, *PS* pressure support, *PEEP* positive end-expiratory pressure, *Crs* total respiratory system compliance

^†^P0.1 defined as the negative pressure measured at the airway opening 100 ms after the initiation of an inspiratory effort, performed against a closed respiratory circuit

Eleven patients (91.7%) underwent PPT prior to PTM/PM detection. PPT was performed twice (1–4) before the initiation of PTX/PM, and 3 days (1–8) had passed since the last PPT. This indicates that many cases had difficulty improving oxygenation.

The complications, PTX management, and outcomes are shown in Table 2.

Two patients who presented with PM were conservatively treated. Signs of PM disappeared in one patient, and neither patient survived. Of the 10 patients with PTX, eight were treated with tube thoracostomy, and two were managed conservatively. Three of the eight patients were unable to remove the chest drain and did not survive. The remaining five patients recovered from PTX, all were able to remove the chest drain. One of them underwent additional pleurodesis. The median duration of chest drainage was 11 days (5–22).

Two patients could not maintain adequate oxygenation after tube thoracostomy and underwent VV-ECMO. One patient underwent VV-ECMO because the respiratory condition deteriorated when PM developed, and the ventilator was set to lung rest settings.

The overall mortality rate for PTX/PM was 50% (6/12). Among the 10 patients who developed PTX, three patients who failed to show radiological improvement did not survive. Seven patients recovered from PTX, only one patient died. These results shows a correlation between PTX treatment failure and mortality.

## Discussion and conclusions

In this case series, we evaluated the clinical characteristics of mechanically ventilated patients with severe COVID-19, who developed PTX/PM. In patients with COVID-19 pneumonia, the complication of PTX/PM is reported to be around 1.7–10% [11]. Our results reported it to be 7.3%, which is in line with the results of the previous reports.

The 12 patients included in our study had difficulty in treatment, as nine patients (75%) required treatment with VV-ECMO, and the length of ICU of stay was 53 days (19–63.5). Reportedly, the mortality rate of patients with ARDS who had PTX was worse than that of patients with ARDS alone [8]. In this study, the mortality rate of patients with PTX/PM was 50%, which is high compared to the mortality rate of 19.4% of all patients who underwent mechanical ventilation in our hospital.

All patients in our study were male, and a multicenter retrospective case series reported that males are 3.3 times more likely to develop PTX/PM than females [2].

Underlying lung diseases, particularly emphysema and interstitial pneumonia, is generally cited as a risk factor for secondary PTX [12]. However, none of the patients in our study had such a history. There have been reports of cases in which bullae were formed that did not present any signs of emphysema on chest CT scans prior to COVID-19 [13]. The same phenomenon occurred in three of our patients, as newly formed bullae were documented in a chest CT scan prior to the development of PTX/PM. The background of these novel bullae formations could be barotrauma due to positive pressure ventilation [2] as well as ischemic parenchymal damage, inflammation, and activation of fibroblasts causing cyst formation [14]. However, our study did not include any patients who underwent pathological evaluation, and the underlying pathology remains unknown.

Patients with COVID-19 can develop severe pneumonia, which can lead to ARDS. Some studies had shown that the proportion of patients with COVID-19 who were diagnosed with ARDS was 42% [15]. PTX is more common in intubated patients with ARDS than in those without ARDS [16]. ARDS lung parenchymal lesions are characterized by heterogeneous distribution of healthy and diseased alveoli around the dorsal side. During high positive pressure ventilation, overdistention of normal lung regions with relatively higher compliance and less airway resistance occurs; thus, alveoli can rupture due to the disproportionate distribution of volume and pressure from the ventilator that causes increased shear forces [17].

The occurrence of PTX in patients with ARDS is most likely associated with the severity of the disease and the longer duration of mechanical ventilation. Gattinoni *et al.* found that the incidence of PTX was higher in patients with ARDS who received mechanical ventilation for a long duration (87% vs. 30% in those with > 2 weeks of mechanical ventilation vs. < 1 week) [17].

In addition, cumulative steroid use may be a potential risk factor for the development of PTX/PM. Steroids used in the treatment of connective tissue diseases, such as dermatomyositis, have been postulated to contribute to the development of spontaneous PM by weakening the pulmonary interstitial tissue, causing alveolar air leak [18]. In our study, the incidence of PTX was higher in patients who underwent steroid pulse therapy than in those who received standard dexamethasone treatment (10.0% vs. 7.3%), suggesting that a higher dosage of steroids could potentially be a risk factor. However, this could merely reflect the severity of the illness, as pulse methylprednisolone therapy was selected for patients with more severe diseases.

Some cases of PTX/PM occurred after PPT, and the incidence of PTX in intubated patients who underwent PPT was higher than the mechanically ventilated patients (11.3% vs. 7.3%). However, this may reflect the fact that PTX/PM occurred more frequently in patients resistant to conventional mechanical ventilation therapy where PPT was necessary, rather than being the influenced by PPT itself.

Pulmonary barotrauma due to mechanical ventilation is a well-known risk factor of PTX/PM. This particularly occurs in cases of high peak inspiratory pressures (40–50 cmH_2_O), high PEEP, and high tidal volumes [17]. However, a high peak inspiratory pressure of 40–50 cmH2O was not detected in our study population. Cases of PTX/PM have been reported even in the absence of positive pressure ventilation with mechanical ventilation, suggesting that positive pressure is not necessarily a condition for PTX/PM [19].

Some researchers have hypothesized that increased respiratory effort generates severe intrapulmonary strains, resulting in the rupture of alveolar cysts. In this study, we measured respiratory effort using the P0.1. However, this was never strong in our study as none of the patients presented with P0.1 higher than 3.5 to 4.0 cmH2O [20]. Additionally, the respiratory condition just before the occurrence of PTX/PM was not necessarily bad (P/F, 185.5 [140.9–260.8]). Therefore, there was a limited correlation between positive pressure ventilation, strong inspiratory effort, and worsening respiratory condition with the occurrence of PTX/PM in our study cohort.

This study had some limitations. First, this study was analyzed retrospectively with a small sample size. All patients were Japanese, which limits the generalizability of the results. Second, although we performed radiography every day, except during the PPT, it is not certain whether the occurrence of PTX/PM was recognized on time. Also, the minute PM was not necessarily detectable on daily radiography and was detected only after the CT scan. Third, we could not fully consider the effects of bronchoscopy or recruitment maneuvers due to the retrospective nature of our study and incomplete records. The strong negative pressure that could have been applied by these maneuvers could have affected the occurrence of PTX/PM. Fourth, treatment details were partly selected at the discretion of the attending physician, so they were not standardized in all cases. Fifth, our study is a case series for patients with COVID-19, who required mechanical ventilation and developed PTX/PM, and we were unable to compare it with those who did not develop PTX/PM.

In conclusion, we were unable to identify the apparent causative factors of PTX/PM, similar to that in previous reports. However, we found that barotrauma and strong inspiratory efforts may be less involved than mentioned in previous studies. PTX/PM in patients with COVID-19 depends on various factors and is likely to be unpredictable. The mortality rate of PTX/PM in COVID-19 was much higher than that of all patients who underwent mechanical ventilation. Therefore, if PTX/PM can be prevented, the mortality rate for COVID-19 may be greatly reduced. Further research is needed to identify its cause and possible prevention.

## Data Availability

Data and materials of this work are available from the corresponding author on reasonable request.

## Abbreviations

PTX: Pneumothorax
PM: Pneumomediastinum
COVID-19: Coronavirus disease 2019
ICU: Intensive care unit
P/F: PaO_2_/FIO_2_
PPT: Prone position therapy
VV-ECMO: Venovenous extracorporeal membrane oxygenation
ARDS: Acute respiratory distress syndrome
PEEP: Positive end-expiratory pressure
CT: Computed tomography
BMI: Body mass index
